# Cabin air dynamics: Unraveling the patterns and drivers of volatile organic compound distribution in vehicles

**DOI:** 10.1093/pnasnexus/pgae243

**Published:** 2024-07-23

**Authors:** Rui Zhang, Minglu Zhao, Hengwei Wang, Haimei Wang, Hui Kong, Keliang Wang, Petros Koutrakis, Shaodan Huang, Jianyin Xiong

**Affiliations:** School of Mechanical Engineering, Beijing Institute of Technology, Beijing 100081, China; School of Computer Science and Engineering, Macau University of Science and Technology, Macau 999078, China; School of Mechanical Engineering, Beijing Institute of Technology, Beijing 100081, China; School of Mechanical Engineering, Beijing Institute of Technology, Beijing 100081, China; School of Mechanical Engineering, Beijing Institute of Technology, Beijing 100081, China; School of Mechanical Engineering, Beijing Institute of Technology, Beijing 100081, China; Department of Environmental Health, Harvard T.H. Chan School of Public Health, Boston 02115, USA; Department of Environmental Health, Harvard T.H. Chan School of Public Health, Boston 02115, USA; Department of Occupational and Environmental Health Sciences, School of Public Health, Peking University, Beijing 100191, China; School of Mechanical Engineering, Beijing Institute of Technology, Beijing 100081, China

**Keywords:** Volatile organic compounds, vehicle cabin environment, attention mechanism, long short-term memory network, deep learning

## Abstract

Volatile organic compounds (VOCs) are ubiquitous in vehicle cabin environments, which can significantly impact the health of drivers and passengers, whereas quick and intelligent prediction methods are lacking. In this study, we firstly analyzed the variations of environmental parameters, VOC levels and potential sources inside a new car during 7 summer workdays, indicating that formaldehyde had the highest concentration and about one third of the measurements exceeded the standard limit for in-cabin air quality. Feature importance analysis reveals that the most important factor affecting in-cabin VOC emission behaviors is the material surface temperature rather than the air temperature. By introducing the attention mechanism and ensemble strategy, we present an LSTM-A-E deep learning model to predict the concentrations of 12 observed typical VOCs, together with other five deep learning models for comparison. By comparing the prediction–observation discrepancies and five evaluation metrics, the LSTM-A-E model demonstrates better performance, which is more consistent with field measurements. Extension of the developed model for predicting the 10-day VOC concentrations in a realistic residence further illustrates its excellent environmental adaptation. This study probes the not-well-explored in-cabin VOC dynamics via observation and deep learning approaches, facilitating rapid prediction and exposure assessment of VOCs in the vehicle micro-environment.

**Significance Statement**

Realistic vehicle cabin study shows that material surface temperature crucially influences the in-cabin VOC emission characteristics, rather than air temperature. This particularly affects new vehicles in summer hot days, explaining the strong new car smell. Our research adopts field measurements on a new car, and a deep learning model with attention mechanism and ensemble strategy to accurately predict in-cabin VOC concentrations. This approach addresses challenges in traditional physics-based models, especially in varied in-cabin conditions, offering advancements in smart commuting and dynamic VOC analysis.

## Introduction

“New car smell” has garnered significant attention from the general populace (1). Volatile organic compounds (VOCs) are the primary contributors to the new car smell, predominantly originated from the emissions of materials in vehicle cabins (2). These emissions have discernible implications for both the in-cabin air quality and human health (3–5). Similar to the effects of indoor VOCs on people, VOCs emitted from materials in vehicle cabins can cause headaches, inflammation of the eyes, nose and throat, fatigue, irritability, dry cough, lung disease, and even disorientation of the driver, which are known as sick car syndrome (6–9). High temperature positively impacts the VOC emissions and will increase the exposure risks. Several teams have independently verified that July 2023 marked the hottest month on record, and there is more to come (10). As the hottest summer descends upon us, the VOC emissions in enclosed spaces, particularly in the primary mode of personal transportation, namely cars, becomes an indispensable attention. Approximately 90% of a person's life is spent indoors, with 5.5% of that time being in cars (11). Hence, there exists a pressing imperative to investigate the emission characteristics of VOCs in cars, aiming to assess the in-cabin air quality and ensure the comfort and health of occupants. Nevertheless, the intricacy and dynamics of actual in-cabin environment make direct prediction and monitoring of the temporal shifts in air pollution challenging (12). This subject bears relevance for enhancing the comfort and health of drivers and passengers, advancing smart car technologies, and conducting exposure assessments.

Physical models employed to describe the emission characteristics of VOCs in indoor and in-cabin environments rely on three key parameters: the initial emittable concentration (*C*_0_), the diffusion coefficient (*D*_m_), and the partition coefficient (*K*) (13, 14). Previous researches have yielded comprehensive exploration and advancement in both the domain of the physical models and the methodologies employed for the determination of key parameters. The physical significance of the three key parameters was firstly clarified in a single-layer emission model proposed by Little et al. (15). A multitude of subsequent studies have further refined the understanding by altering the layers within the physical model, increasing the quantity of emission sources, and manipulating the conditions of emission environment (16–23). The measurement methods for these parameters are diverse, encompassing but not confined to approaches such as the twin chamber method, the extraction method, the C-history method, and the nonlinear regression method (24–30). Nevertheless, models and methodologies for characterizing actual in-cabin VOCs remain largely underexplored in prior researches. The realistic vehicle cabin environment hosts a diverse array of emission sources (seats, carpets, ceilings, etc.), each fulfilling multifaceted roles that span from being mere emitters to simultaneous emitters and absorbers. Additionally, the spatial distribution of these sources further increases the complexity. These combined intricacies intensify the difficulties and uncertainties associated with utilizing conventional physical models for prediction. As a result, efforts to devise accurate and fast approaches to the actual in-cabin environment are urgently needed, although marked by formidable challenges.

Environmental investigation is inherently complex and often involves large amounts of mineable data (31). Deep learning, a branch of machine learning, stands as one of the most recent trends within the realm of artificial intelligence (12). For artificial intelligence, simple recurrent neural network (RNN), long short-term memory (LSTM), gated recurrent unit (GRU), convolutional neural network (CNN), and CNN-LSTM models indicate widespread application in atmospheric environment for the prediction of air pollutants such as PM_2.5_, PM_10_, SO_2_, ozone, and more (32–37). Recent usage in weather prediction elucidates the prospects and potential of artificial intelligence in environmental fields (38). Some researchers have conducted investigations on pollutants in indoor environments. Loy-Benitez et al. (39) employed RNN, LSTM, and GRU to achieve point-by-point predictions of PM_2.5_ levels in subway stations with acceptable accuracy. Lagesse et al. (40) applied different machine learning models to predict PM_2.5_ levels in large office buildings and concluded that LSTM model was the best. Nurcahyanto et al. (41) conducted a study on indoor air quality by employing RNN and LSTM models for long-term prediction of PM_10_ concentration, humidity, and temperature in a cleaning room, and found that the multilayer RNN model outperformed the LSTM model. Yuan and Yang (42) introduced a hybrid model comprising a self-attention mechanism, empirical mode decomposition algorithm, and LSTM, and successfully achieved prediction of PM_2.5_ concentrations in classrooms. Zhang et al. made multiple attempts to apply artificial neural network models (43), single-feature LSTM model (44), and multifeature LSTM model (45) to forecast VOC concentrations emitted from furniture, classroom, and actual residence, with acceptable outcomes. Deep learning models, including RNN and LSTM models, have gained recognition for their efficacy in predicting indoor pollutant concentrations, and are also playing an important role in controlling the internal environment of smart farming facilities (46, 47). As an inseparable component of human living environment, the in-cabin environment has also captured great attention recently. Goh et al. (48) utilized three machine learning models to predict the in-cabin air quality, with support vector regression emerging as the top-performing predictor. He et al. (49) established a data-driven model for predicting thermal comfort of passengers in each seating position, which helps to the development of personalized thermal comfort in cars. However, the aforementioned studies primarily focus on either atmospheric environments, or specific pollutants such as particulate matter and CO_2_, or thermal comfort in vehicle cabins. Research dedicated to the transport characteristics of VOCs in vehicle cabins via deep learning, the indispensable micro-environment for commuting, has remained unexplored.

The objectives of this study are to: (i) conduct field measurements of VOC emissions in an actual new car during summer time with high-temperature conditions; (ii) employ machine learning techniques to investigate the impact of in-cabin environmental factors (material surface temperature (MST), in-cabin relative humidity (IRH), in-cabin air temperature (IAT), air exchange rate (AER)) on VOC emissions; (iii) by incorporating attention mechanism and ensemble strategy into LSTM, develop an LSTM-A-E model to realize the prediction of typical in-cabin VOC concentrations and validate its application for other indoor environments.

## Results and discussion

### Measured environmental parameters for the tested car

To assess the emission characteristics of VOCs from materials in cabin environment under varying summer weather conditions, experiments were conducted in an unmanned car with the engine turned off. Our measurements encompassed the use of a CO_2_ tracer method for measuring the AER (50), a multiplex temperature meter for tracking the in-cabin MST and out-cabin air temperature. Furthermore, we employed a temperature and relative humidity data logger to record IAT and relative humidity levels.

The CO_2_ tracer method involved placing dry ice inside the car and subsequently closing the car doors to maintain experimental conditions. Two CO_2_ concentration meters was concurrently used to measure the concentrations both inside and outside the car. By applying the principles of tracer method, a fitting procedure is employed on the acquired CO_2_ concentration data from both inside and outside the car to deduce the AER when the car is stationary, as indicated by equation (1):


(1) $$\ln\;(C-C_{\mathrm{out}})-\ln\;(C_{\max}-C_{\mathrm{out}})=-Q/V*t$$

where *C* and *C*_out_ are the in-cabin and out-cabin CO_2_ concentrations, respectively, ppm; *C*_max_ is the CO_2_ concentration at *t* = 0, ppm; *Q/V* represents the AER, h^−1^; *t* is the CO_2_ concentration decay time, h.

We measured the in-cabin AER during the experimental period using the CO_2_ tracer method. Based on the slope of fitted curve with equation (1), we divided the AER into four segments, as shown in Fig. S1. These four segments of AER are 0.184 h^−1^, 0.199 h^−1^, 0.211 h^−1^, and 0.218 h^−1^. The car's AER does not vary greatly due to the close of the car windows and ventilation system.

**Fig. 1.**
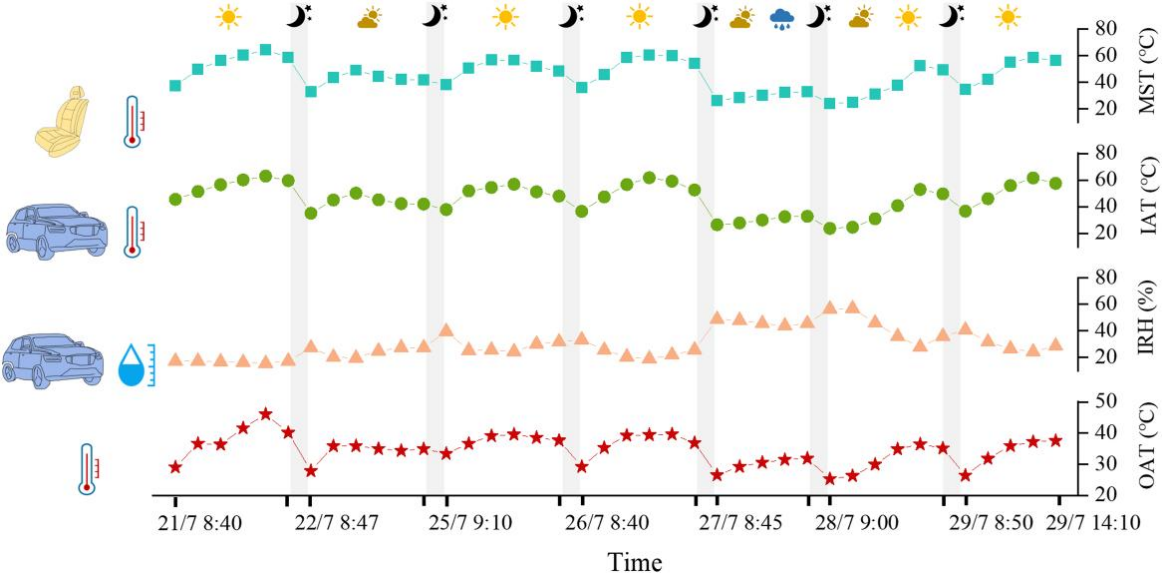
Measured material surface temperature, in-cabin air temperature, in-cabin relative humidity, and out-cabin air temperature during the field tests. MST, material surface temperature; IAT, in-cabin air temperature; IRH, in-cabin relative humidity; OAT, out-cabin air temperature.

Figure 1 shows the measured MST, IAT, IRH, and out-cabin air temperature (OAT) during the tests. Notably, the MST inside the car represents the average temperature across the five seats. This figure reveals that the weather conditions during the experiments encompassed sunny, cloudy, and rainy days, representing typical occurrences in daily life. The fluctuation patterns of MST align with those of IAT and OAT. Furthermore, it is noteworthy that MST exhibits a variable range spanning from 24.1°C to 64.2°C, while IAT ranging from 23.8°C to 63.0°C. The variation in OAT falls within the range of 25.3°C to 46.1°C, a result that marginally exceeds the temperature data provided by the meteorological bureau for that region. This deviation may be attributed to the presence of some surrounding buildings in the vicinity of the testing place, potentially hindering localized air circulation. The trend of IRH is opposite to that of the three temperatures, and the IRH ranges from 15.6 to 56.8% during the entire experiment.

### Observation and analysis of the in-cabin VOC concentrations

**Fig. 2.**
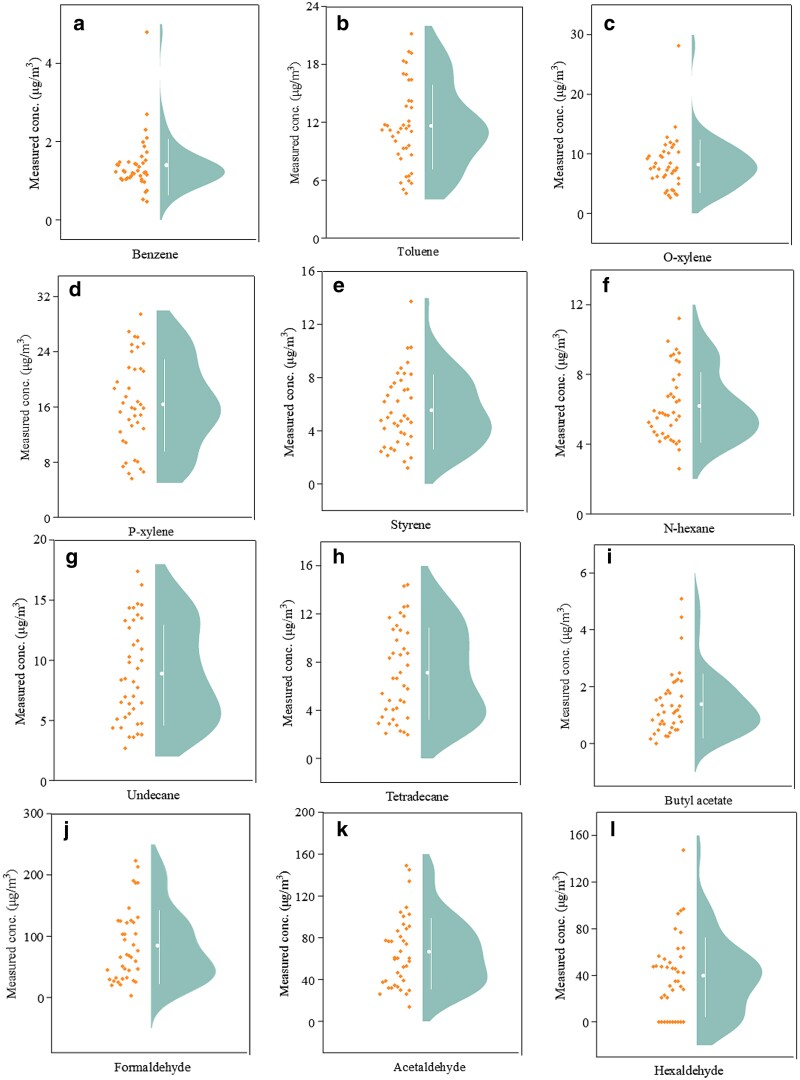
Measured VOC concentrations for 7 days in the actual car cabin. (a) Benzene; (b) Toluene; (c) O-xylene; (d) P-xylene; (e) Styrene; (f) N-hexane; (g) Undecane; (h) Tetradecane; (i) Butyl acetate; (j) Formaldehyde; (k) Acetaldehyde; (l) Hexaldehyde.

For a new car, the interior materials are the main sources of in-cabin air pollution. These materials include plastics, rubber, synthetic fibers, textiles, leather, padding materials, adhesives, etc. They can emit varying amounts of VOCs during their usage. For this study, a comprehensive 7-day measurement in a new car yielded concentration data for 12 common VOCs, with 3 of them belonging to the aldehyde category, while the remaining 9 VOCs encompassed a diverse array of compounds, excluding aldehydes. Figure 2 illustrates the concentration distribution in the actual car cabin over the tested period. Figure 2(a)-(e) shows the concentration distribution of benzene and benzene series. It can be seen that most of the benzene concentrations are concentrated at 1.5 μg/m^3^, a small portion of them are distributed in the range of 1.0–3.0 μg/m^3^, and only one point has a concentration of 4.8 μg/m^3^, indicating that benzene emission is in a relatively low level. The concentration distribution of o-xylene is similar to that of benzene, with the majority falling within the range of 5.0–15.0 μg/m^3^. The distribution patterns suggest that changes in environmental factors have relatively little impact on benzene and o-xylene. The concentration distributions of toluene, p-xylene, and styrene exhibit similarities, with measurement points falling almost uniformly within their respective concentration ranges (5–20 μg/m^3^, 5–30 μg/m^3^, and 1–10 μg/m^3^). This indicates that the concentrations of these three benzene series are easily affected by environmental factors and should be considered in future studies. Among the five benzene series in Fig. 2(a)-(e), the mean concentration of p-xylene is the highest, at 16.4 μg/m^3^. The benzene concentration levels in car cabin are higher than those in residential air, and it should be noted that, benzene, a class I carcinogen identified by the WHO, has no specific guidance value (51).

Figure 2(f)-(h) shows the concentration distribution of alkanes, n-hexane, undecane and tetradecane, which are similarly distributed, with a high number of measurement points distributed in both the low and high concentration regions. The concentration distribution of butyl acetate is depicted in Fig. 2(i), with its concentration primarily centered around 1.5 μg/m^3^. Figure 2(j)-(l) illustrates the concentration distributions of formaldehyde, acetaldehyde, and hexaldehyde. These three aldehydes exhibit wide concentration ranges and are susceptible to environmental factors. Of greater significance is the fact that their concentrations markedly surpass those of the above-mentioned 9 VOCs. Formaldehyde concentrations are mainly distributed within the range of 30 to 130 μg/m^3^, and some even exceeding 200 μg/m^3^. The Chinese national standard GB/T 27630–2011 prescribes a concentration limit of 100 μg/m^3^ for formaldehyde in vehicle cabins (52). Among the 40 measurement points recorded over the 7 tested days, 37.5% (15 measurements) exceeds this specified limit. Moreover, formaldehyde concentration ranks the highest among the 12 VOCs monitored. These observations collectively emphasize the undeniable significance of formaldehyde in the study of in-cabin air pollution. The concentration of acetaldehyde is also noteworthy. Figure 2(k) indicates that 30–100 μg/m^3^ is the main concentration interval of acetaldehyde distribution, and there are a few measurement points that can reach 140 μg/m^3^, while the limit value is 50 μg/m^3^ (52). In total, there are 25 measurement points whose concentrations exceed the limit. The concentration of hexaldehyde seems to exhibit a heightened vulnerability to environmental factors, as evidenced by the weather on July 27, which experienced a shift from cloudy to rainy conditions accompanied by low temperature and high humidity. Remarkably, the hexaldehyde concentration remains approximately 0 μg/m^3^ throughout this day, while for most of other days, it ranges from 30 to 100 μg/m^3^. Consequently, it is crucial for the drivers and passengers to pay attention to hexaldehyde emissions, particularly during periods of elevated temperatures.

### Impact of in-cabin environmental factors on VOC concentrations in the car

Multiple factors can influence the material emission characteristics (emission rate or concentration), including temperature, relative humidity, and AER in the space where the materials are located. In this section, the impact of in-cabin environmental factors (MST, IAT, IRH, and AER) on VOC emissions is explored through machine learning methods. Feature selection can preserve crucial attributes, diminish extraneous factors and computational burdens, and enhance the efficiency of model learning (53). Feature importance analysis, a vital technique for machine learning/deep learning feature selection, assesses input variable impact on the target variable. It can offer a strategic input variable selection, maximizing cost-effectiveness. This will help to better control the in-cabin VOC pollution by grasping the main contributors.

**Fig. 3.**
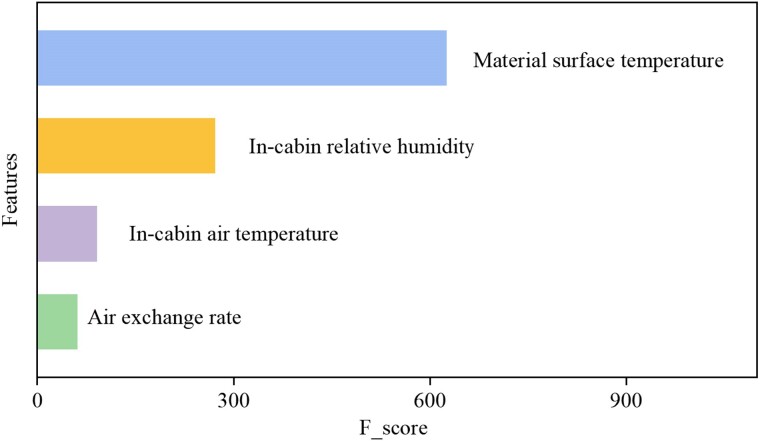
Impact of four in-cabin environmental factors on VOC emission characteristics. Toluene is taken as an example for analysis; F_score represents the number of split points for every feature.

Figure 3 shows the impact of four in-cabin environmental factors on VOC emission behaviors. We used feature importance analysis, a common machine learning method ranking features by their F_score. MST emerges as the key factor affecting VOC concentration, surpassing IAT, IRH, and AER. In prior studies, researchers mainly focused on air temperature on material emissions, while neglecting the impact of MST. This ignorance may lead to model prediction discrepancies. Figure 3 reiterates the results obtained by Wang et al.'s (23) findings using the conventional physical model.

IRH and IAT are the subsequent factors affecting VOC concentrations in the car. The importance of IRH falls just below that of MST, potentially attributed to the weather conditions during experiment. This study encompassed diverse weather scenarios, including sunny, cloudy, and rainy conditions. AER ranks the lowest of all the influencing factors, contrary to the conclusion of exploring the influencing factors of VOC concentrations in residence (where AER was the most important influencing factor) (45). This is understandable, since during the experiment, both the car doors and windows were closed, and the engine was turned off, thus only the infiltration could influence the AER. While in occupied residence, factors such as opening and closing of windows and doors come into play, leading to more fluctuations in AER, and subsequently a noticeable impact on indoor VOC concentrations. This finding opens up diverse avenues for further, in-depth examinations of VOC emissions in actual car cabins across different operational scenarios and environmental conditions. It also offers tailored directions for reducing VOC concentrations in practical situations.

### Prediction of VOC concentrations in the car cabin via deep learning

The in-cabin VOC concentrations greatly affect the comfort of driver and passenger. Predicting VOC concentrations accurately and swiftly is crucial. With technological advancements and societal changes, car intelligence is booming. Besides autonomous driving in AI, researchers now focus on using AI to improve in-cabin comfort. However, most efforts prioritize the impact of temperature and humidity on occupant comfort (49), while neglecting VOCs. In view of this, we built an LSTM-A-E model to predict the in-cabin VOC concentrations (the principle of the developed model is introduced in the “Deep learning models” section), together with the fully connected neural network (FNN), RNN, GRU, LSTM and LSTM-A models for comparison. To compare their performance, we maintained consistent parameters, and the setup of parameters for these models are listed in Table S1. We selected all measured physical parameters as inputs for our model: MST, IAT, IRH, AER, and OAT, with VOC concentration as the target variable. In the 7-day field experiment (July 21–22, 25–28), the model identified VOC emission patterns from the first 6 days, using them for learning. The model's performance was validated on the 7^th^ day (July 29). No experiments were conducted on the July 23–24 days due to scheduled rest periods.

**Fig. 4.**
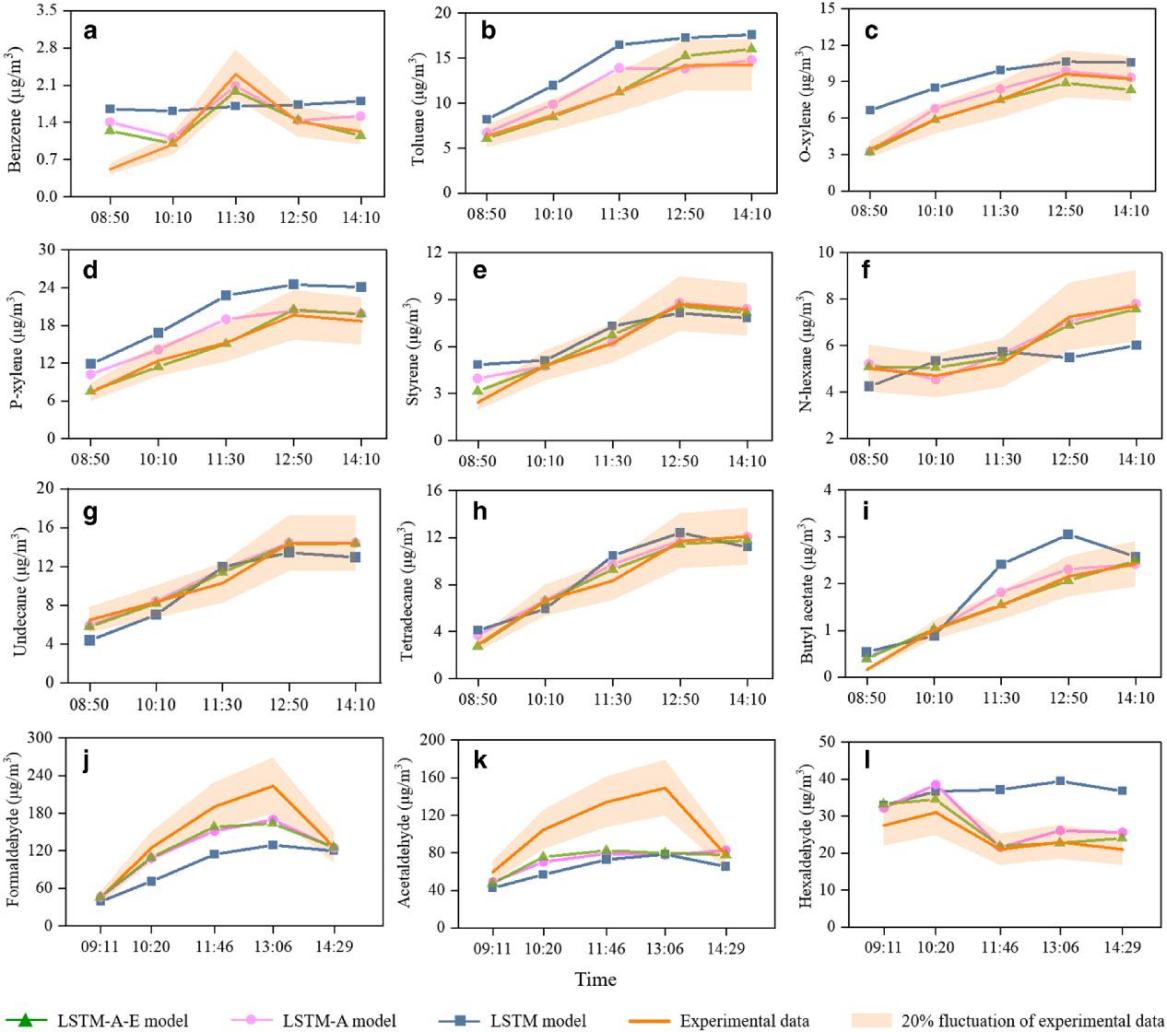
The predicted VOC concentrations by LSTM-A-E, LSTM-A, and LSTM models, and comparison with experimental data. (a) Benzene, (b) Toluene, (c) O-xylene, (d) P-xylene, (e) Styrene, (f) N-hexane, (g) Undecane, (h) Tetradecane, (i) Butyl acetate, (j) Formaldehyde, (k) Acetaldehyde, and (l) Hexaldehyde.

Figure 4 presents the results for benzene, toluene, o-xylene, p-xylene, styrene, n-hexane, undecane, tetradecane, butyl acetate, formaldehyde, acetaldehyde, and hexaldehyde prediction using LSTM, LSTM-A, and LSTM-A-E models. (To make the figure clear, the results of other three models are not shown in this figure, but given in the following Table 1.) This figure indicates that, for the benzene prediction (Fig. 4(a)), the LSTM-A-E model closely follows the trend of the experimental data, with a substantial portion residing within the orange band. This observation signifies the superior predictive efficacy of the LSTM-A-E model compared to the LSTM and LSTM-A models, which exhibits challenges in capturing variations in benzene concentration and is thus ill-suited for benzene concentration prediction. For toluene, o-xylene, p-xylene, and styrene (Fig. 4(b)-(e)), both models demonstrate the capability to capture changes in concentration and uncover hidden patterns within the concentration data. It is evident that the LSTM-A-E model consistently yields predictions that closely approximate experimental data when compared to the LSTM and LSTM-A models. The predictions by the LSTM-A-E model mainly fall within the orange band, signifying that prediction errors consistently remain below 20%, indicating a favorable predictive performance. The predictive results for n-hexane, undecane, and tetradecane are presented in Fig. 4(f)-(h). As observed in the figures, for alkanes, the three models generally produce similar and satisfactory predictions. However, in some scenarios, such as n-hexane (Fig. 4(f)) at 12:50 AM and 2:10 PM, the LSTM-A and LSTM-A-E models demonstrate a clear superiority, potentially owing to its enhanced capacity for capturing fluctuations in VOC concentrations by the incorporation of an attention mechanism.

**Table 1.** SSE, RMSE, NMSE, MAE, and MAPE values of the six models (FNN, RNN, GRU, LSTM, LSTM-A, and LSTM-A-E) for predicting 12 VOC concentrations.

VOCs	Metrics	FNN	RNN	GRU	LSTM	LSTM-A	LSTM-A-E
Benzene	SSE	3.397	2.187	2.436	2.461	0.923	0.627
	RMSE	0.824	0.661	0.698	0.702	0.430	0.354
	NMSE	0.488	0.207	0.217	0.224	0.095	0.071
	MAE	0.687	0.591	0.654	0.649	0.306	0.232
	MAPE	66.8%	69.4%	75.3%	75.1%	43.1%	32.3%
Toluene	SSE	32.791	37.042	61.687	62.772	8.856	4.245
	RMSE	2.561	2.722	3.512	3.543	1.331	0.921
	NMSE	0.046	0.050	0.079	0.080	0.014	0.007
	MAE	2.223	2.503	3.352	3.366	1.008	0.668
	MAPE	25.4%	26.5%	33.1%	31.8%	9.7%	5.5%
O-xylene	SSE	21.144	71.540	60.473	25.598	1.589	1.497
	RMSE	2.056	3.783	3.478	2.263	0.564	0.547
	NMSE	0.067	0.190	0.161	0.078	0.006	0.006
	MAE	1.671	3.423	3.409	2.114	0.451	0.411
	MAPE	34.7%	63.2%	55.1%	38.9%	7.1%	5.4%
P-xylene	SSE	104.181	158.349	157.921	148.209	25.930	2.866
	RMSE	4.565	5.628	5.620	5.444	2.277	0.757
	NMSE	0.075	0.107	0.107	0.101	0.021	0.003
	MAE	4.378	5.515	5.488	5.324	1.984	0.645
	MAPE	36.9%	42.4%	40.9%	39.9%	17.0%	4.2%
Styrene	SSE	10.706	11.248	8.032	7.684	2.256	0.803
	RMSE	1.463	1.500	1.267	1.240	0.672	0.401
	NMSE	0.055	0.057	0.040	0.038	0.012	0.004
	MAE	1.285	1.270	1.018	0.989	0.344	0.322
	MAPE	31.9%	33.0%	27.6%	27.2%	13.0%	8.3%
N-hexane	SSE	10.639	6.566	6.264	7.226	0.270	0.330
	RMSE	1.459	1.146	1.119	1.202	0.232	0.257
	NMSE	0.049	0.035	0.037	0.045	0.001	0.002
	MAE	1.199	0.982	1.092	1.073	0.213	0.226
	MAPE	22.2%	18.3%	18.3%	17.0%	3.8%	3.9%
Undecane	SSE	11.153	12.281	9.221	11.797	2.280	1.658
	RMSE	1.494	1.567	1.358	1.536	0.675	0.576
	NMSE	0.019	0.023	0.016	0.022	0.004	0.003
	MAE	1.393	1.452	1.118	1.487	0.419	0.415
	MAPE	13.5%	13.8%	12.2%	16.1%	4.7%	4.9%
Tetradecane	SSE	21.368	16.651	9.158	7.941	2.462	1.112
	RMSE	2.067	1.825	1.353	1.260	0.702	0.472
	NMSE	0.063	0.042	0.024	0.022	0.007	0.003
	MAE	1.659	1.491	1.123	1.143	0.445	0.373
	MAPE	30.4%	26.8%	18.6%	18.4%	8.8%	4.9%
Butyl acetate	SSE	1.252	1.021	3.480	1.759	0.175	0.064
	RMSE	0.500	0.452	0.834	0.593	0.187	0.113
	NMSE	0.129	0.080	0.218	0.128	0.015	0.006
	MAE	0.396	0.416	0.733	0.487	0.146	0.081
	MAPE	89.2%	90.4%	102.9%	69.6%	38.8%	29.3%
Formaldehyde	SSE	17,475.239	20,498.719	16,048.235	17,607.162	4677.958	5065.606
	RMSE	59.119	64.029	56.654	59.342	30.587	31.830
	NMSE	0.250	0.312	0.225	0.262	0.055	0.060
	MAE	46.720	50.794	43.293	47.082	22.284	22.251
	MAPE	28.6%	29.6%	25.1%	28.5%	12.2%	11.8%
Acetaldehyde	SSE	12,501.768	14,218.494	11,972.981	11,418.163	9350.254	8586.377
	RMSE	50.004	53.326	48.935	47.787	43.244	41.440
	NMSE	0.378	0.462	0.357	0.344	0.251	0.227
	MAE	41.807	46.209	41.011	41.695	34.313	32.766
	MAPE	35.4%	40.5%	35.0%	36.6%	28.1%	27.0%
Hexaldehyde	SSE	2,805.376	3,543.610	2,987.231	840.646	110.328	53.420
	RMSE	23.687	26.622	24.443	12.966	4.697	3.269
	NMSE	0.523	0.583	0.511	0.186	0.031	0.016
	MAE	21.072	24.490	22.671	11.894	4.113	2.631
	MAPE	93.4%	107.7%	98.9%	52.3%	16.0%	10.2%

SSE, The sum of squares due to error (μg/m^3^)^2^; RMSE, Root mean squared error (μg/m^3^); NMSE, Normalized mean square error; MAE, Mean absolute error (μg/m^3^); MAPE, Mean absolute percentage error.

Figure 4(i) illustrates the predictions of three models for butyl acetate. The discernible observation from the figure is that, the LSTM-A-E model outperforms, with prediction errors within 20% for all measurement points, except the first one. As evident from Fig. 4(j)-(l), the LSTM model exhibit suboptimal performance for formaldehyde, acetaldehyde, and hexaldehyde, with all prediction points broadly falling outside the orange band. Particularly for hexaldehyde, the LSTM model fails to capture the emission trend, resulting in relatively large discrepancy under varying temporal and environmental conditions. This finding highlights the necessity of strengthening the learning capacity of LSTM model in learning the emission patterns of aldehydes concentrations in actual car cabins. For both formaldehyde and hexaldehyde, the LSTM-A-E model outperforms the LSTM and LSTM-A models, with prediction errors averaging around 20%. As for acetaldehyde, neither of these three models demonstrate satisfactory capabilities. This limitation may be attributed to the complexity of the underlying emission patterns associated with acetaldehyde, rendering it difficult for both deep learning models to effectively learn information from the concentration data. This implies that for the prediction of the concentration of aldehydes (especially acetaldehyde) in actual cars, we need to increase the amount of data and complexity of model learning to ensure that the model can cope with the prediction of multiple VOC concentrations. The above analysis demonstrates the advantages of the constructed LSTM-A-E model in the prediction of VOC emission characteristics in actual car cabins.

Evaluation metrics provide a more intuitive means of assessing the overall predictive performance of the six deep learning models. Table 1 gives the sum of squares due to error (SSE), root mean squared error (RMSE), normalized mean square error (NMSE), mean absolute error (MAE), and mean absolute percentage error (MAPE) for the prediction of 12 VOC concentrations by the six deep learning models. For the five benzene compounds, the performance of the FNN, RNN, GRU, and LSTM models are generally subpar, while the LSTM-A model exhibits excellent capability, with MAPE values below 10% for toluene and o-xylene and within 20% for p-xylene and styrene. LSTM-A-E model shows the best performance, and MAPE values of toluene, o-xylene, p-xylene, and styrene are all less than 10%. LSTM-A and LSTM-A-E models exhibit favorable performance for three compounds (n-hexane, undecane, tetradecane), with MAPE values consistently below 10%. Based on the SSE, RMSE, NMSE, MAE, and MAPE metrics, the FNN, RNN, GRU, and LSTM models exhibit suboptimal prediction ability for formaldehyde, acetaldehyde, and hexaldehyde concentrations in actual car cabins. The LSTM-A and LSTM-A-E models outperform in predicting formaldehyde and hexaldehyde, achieving MAPE values below 20%. In particular, LSTM-A-E model is significantly better than other models in predicting hexaldehyde. By combining the results in Fig. 4 and Table 1, the LSTM-A-E model proves promising for short-term in-cabin VOC concentration prediction.

### Application of the model in predicting VOC concentrations in an actual residence

To examine the adaptation of the LSTM-A-E model in different realistic indoor settings, this model is extended to predict the VOC concentrations in a real residence that we had conducted field campaign previously (53–56). This study focused on the compound of acrolein, utilizing data collected over a span of 15 days, from 2016 August 24 to 29, for training the LSTM-A-E model. Furthermore, we employed data spanning 10 days, from August 31 to September 9, as a validation dataset to evaluate the model's performance. The predictive results of the LSTM model were reported in a prior publication (45).

Figure S2 shows the predictions of 10-day acrolein concentrations and RD using the LSTM, LSTM-A, and LSTM-A-E models. During the experiment, there were obvious variations in the concentration of acrolein in the residence, particularly at 12:50 AM on September 3 and at 5:50 PM on September 7. Previous investigation (45) indicated that the significant peaks in concentration at these two moments were attributed to human activities. Figure S2 indicates that both models closely match experimental trends, affirming their ability to capture acrolein emission patterns. The LSTM-A-E model consistently performs better, highlighting the enhancement of attention mechanism and ensemble strategy for data information learning. To further compare the performance of LSTM, LSTM-A, and LSTM-A-E models, we assess the SSE, RMSE, NMSE, MAE, and MAPE (listed in Table S2). Table S2 shows that the SSE, RMSE, NMSE, MAE, and MAPE of LSTM-A-E model decrease by 14.865 ppb^2^, 0.047 ppb, 0.027, 0.054 ppb, and 4.7% compared with LSTM model. This result illustrates that the LSTM-A-E model is specialized in capturing complex, dynamic emission patterns, showcasing its potential in real-world smart homes.

### Impact of measurement uncertainty on the model performance

**Fig. 5.**
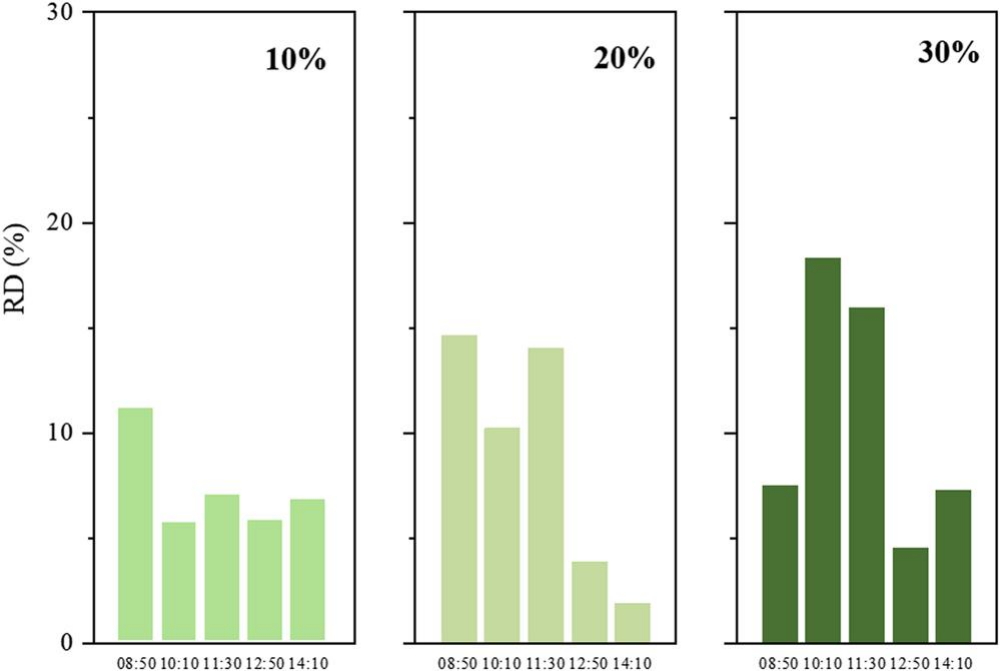
Relative deviation (RD) of LSTM-A-E model prediction with experimental data of toluene at different measurement uncertainties.

Measurement uncertainty poses significant impact on model accuracy, reliability, and data interpretation. Incorporating uncertainty analysis ensures robust conclusions, enhances cross-study comparability, and advances scientific credibility. The precision of the experimental instruments is presented in Table S3. The devices used for temperature and relative humidity measurements exhibit high levels of accuracy. The measurement uncertainty of the GC/MS and HPLC system in the actual car cabins can be regarded as less than 25% (22). Here we analyze the impact of measurement uncertainty on the model performance. Three measurement uncertainties, i.e. 10%, 20%, 30%, are randomly added to the measured concentration data by using a random function. The LSTM-A-E model is used to predict the toluene concentration at five time points on July 29, and Fig. 5 illustrates the RD in the predictions. The RD values for the three uncertainties are all below 20%. Moreover, we calculate MAPE for 10%, 20%, and 30% uncertainties, yielding results of 7.2%, 8.9%, and 10.7%, respectively. This analysis demonstrates that the LSTM-A-E model can perform well even with a 20% or 30% measurement uncertainty in experimental data.

In this study, we presented a deep learning model based on attention mechanism and ensemble strategy for the prediction of VOC concentrations in a realistic car cabin. We conducted measurements of MST, IAT, IRH, and OAT on 7 workdays during various summer weather conditions. The distribution patterns of concentrations for 12 in-cabin VOCs were characterized. The concentration ranges of formaldehyde, acetaldehyde, and hexaldehyde were broad and susceptible to environmental influences, which were significantly higher than the other 9 VOCs. Feature importance analysis with machine learning methodology was served as a valuable tool for evaluating the contributors of in-cabin VOC emissions. The MST, rather than the commonly considered IAT, was found to be the most influential factor on VOC emissions. Six deep learning models, including FNN, RNN, GRU, LSTM, LSTM-A, and LSTM-A-E models, were used for the prediction of 12 actual in-cabin VOC concentrations, with results indicating that the LSTM-A-E model is more promising in the prediction of in-cabin VOC concentrations. Further analysis demonstrated the excellent performance of LSTM-A-E model in environmental adaptation, as well as with large measurement uncertainties. Compared to conventional physical examinations, where the processes of modeling and determining characteristic parameters are complex and difficult, the exploration by LSTM-A-E is simple to model, responsive, and easy to be embedded into the control system of intelligent cars. This, in turn, will promote in-cabin concentration prediction and exposure assessment.

## Methods and materials

### Deep learning models

FNN model is a classic deep learning model, where its information transfer is one-way, and the output of the network only depends on the current input. In practical scenarios, the network's output is contingent on both past outputs over a certain duration and the current input. RNNs, including simple RNN and neural network equipped with gating mechanisms, such as GRU and LSTM, not only receive information from other neurons, but also incorporate information from their own recent history, thereby achieving a degree of memory capability. Nevertheless, in dealing with long-term dependency relationships within lengthy sequences, the RNN mechanism encounters limitations in terms of memory or network capacity, resulting in a swift performance decent as the length of input information grows. Long-range information is attenuated, akin to individuals with weak memory struggling to recollect past events. To mitigate the bottleneck arising from the transformation of long sequences into fixed-length vectors, attention mechanisms (Details are given in Text S1 of the Supplementary material) have been incorporated. Recently, attention mechanism has gained widespread application across various domains in deep learning, from image processing (57) and speech recognition (58), to various types of tasks in natural language processing (59).

**Fig. 6.**
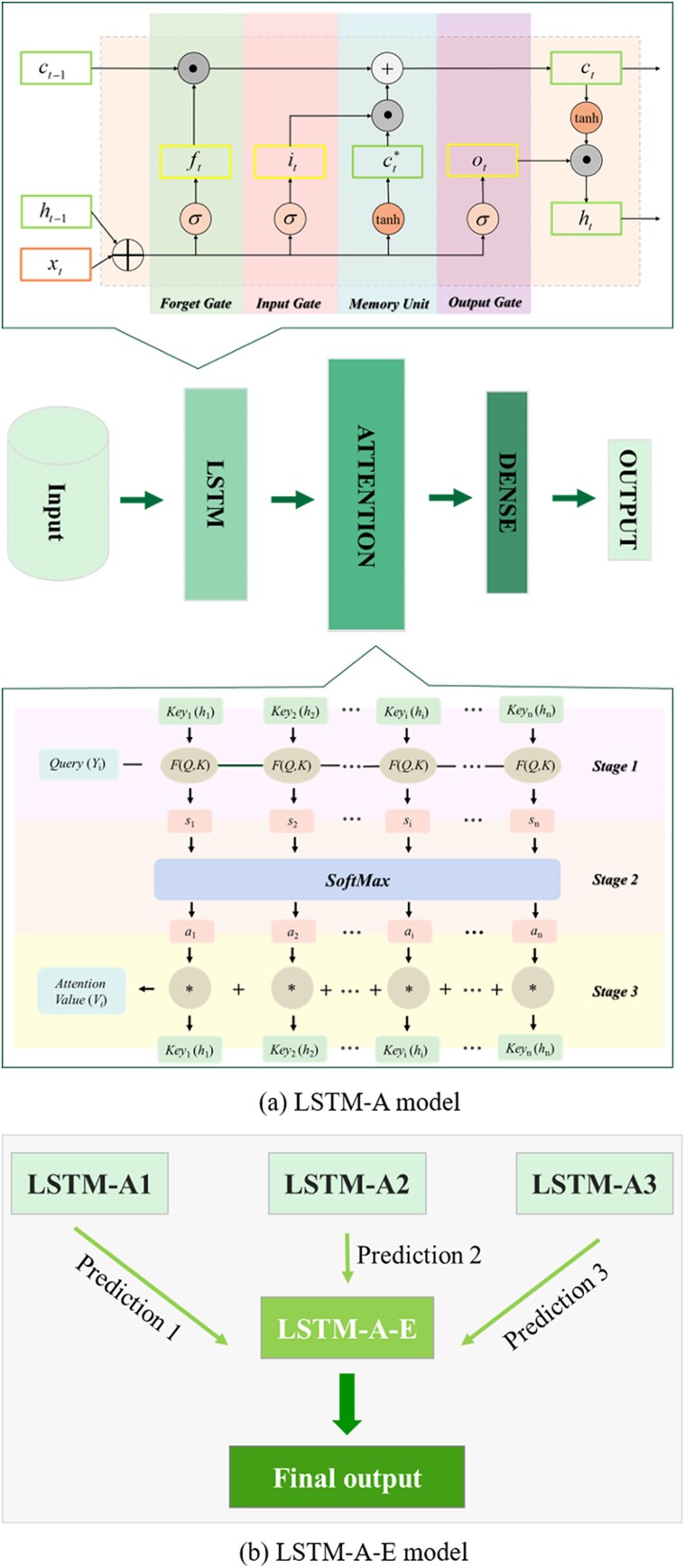
Structures of the LSTM-A model and LSTM-A-E model. (a) LSTM-A, LSTM network based on attention mechanism; (b) LSTM-A-E, integrated model for three LSTM-A models.

Figure 6(a) illustrates the structure of an LSTM network integrated with an attention mechanism (called LSTM-A model). As shown in the figure, the input information proceeds from the input layer into the LSTM layer, passing through the forget gate, input gate, memory unit, and output gate within the LSTM layer before progressing into the attention layer. The information transmission within the attention layer can be divided into three sequential stages. The first stage entails the computation of similarity scores through the evaluation of the similarity between queries and keys. The second stage is to normalize the similarity score by SoftMax function. Finally, the weighted summation of values using the normalized similarity scores yields the attention value (60). The LSTM-Attention-Ensemble (LSTM-A-E) model, derived from the LSTM-A framework, integrates LSTM-A1, LSTM-A2, and LSTM-A3 models (with different structures and parameters) via averaging method. The LSTM-A-E model can capture information from different aspects in a comprehensive manner, thereby enhancing prediction stability and reducing overfitting. The structure of the LSTM-A-E model is schematically shown in Fig. 6(b). The network parameters of different models used in this study are listed in Table S1. It should be noted that the initial values of these parameters are usually determined by personal experience or default values. And then the parameters are adjusted by comparing model predictions with experimental data to find the best combination of parameters to build a model with good performance.

### Evaluation metrics for the deep learning models

Model evaluation plays a critical role in assessing the efficacy of a model, with distinct evaluation metrics describing different aspects of its performance. We select relative deviation (RD), the sum of squares due to error (SSE), root mean squared error (RMSE), normalized mean square error (NMSE), mean absolute error (MAE), and mean absolute percentage error (MAPE), as the metrics from various perspectives. RD evaluates predictive outcomes, reflecting relative deviations. Dimensionless operations enhance its potential to reveal result credibility. SSE, RMSE, and MAE have units. VOC concentration prediction in this study employs concentration units (μg/m^3^ and ppb) for intuitive disparity comprehension. MAE represents error average, while RMSE is sensitive to outliers. Their comparison detects outliers in predictions. NMSE is suitable for evaluating nonlinear data. MAPE is independent of the prediction scale, so it is reasonable to use it as the metric to evaluate the prediction performance of the whole model (32).

Specifically, for the six metrics, RD, SSE, RMSE, NMSE, MAE, and MAPE, the smaller the value of the metric, the better the performance of the prediction. The definitions of the six metrics are as follows.

1. Relative deviation (RD)
  (2) $$\mathrm{RD}=\left|\frac{C_{\mathrm{predicted}\_i}-C_{\mathrm{actual}\_i}}{C_{\mathrm{actual}\_i}}\right|\times 100\text{\%}$$
2. The sum of squares due to error (SSE)
  (3) $$SSE=\sum_{i=1}^{N}{\left(C_{\mathrm{predicted}\_i}-C_{\mathrm{actual}\_i}\right)}^{2}$$
3. Root mean squared error (RMSE)
  (4) $$\mathrm{RMSE}=\sqrt{\frac{1}{N}\sum_{i=1}^{N}{\left(C_{\mathrm{predicted}\_i}-C_{\mathrm{actual}\_i}\right)}^{2}}$$
4. Normalized mean square error (NMSE)
  (5) $$\mathrm{NMSE}=(\bar{C_{\mathrm{predicted}}-C_{\mathrm{actual}}}{)}^{2}/\left[\left(\bar{C_{\mathrm{predicted}}}\right)*\left(\bar{C_{\mathrm{actual}}}\right)\right]$$
5. Mean absolute error (MAE)
  (6) $$\mathrm{MAE}=\frac{1}{N}\sum_{i=1}^{N}\left|C_{\mathrm{predicted}\_i}-C_{\mathrm{actual}\_i}\right|$$
6. Mean absolute percentage error (MAPE)
  (7) $$\mathrm{MAPE}=\frac{1}{N}\sum_{i=1}^{N}\left|\frac{C_{\mathrm{predicted}\_i}-C_{\mathrm{actual}\_i}}{C_{\mathrm{actual}\_i}}\right|\times 100\text{\%}$$
  where *N* is the number of predictions for each VOC; *C*_predicted_*i*_ is the *i*th predicted concentration by different models; *C*_actual_*i*_ is the *i*th measured concentration;

$(\bar{C_{\mathrm{predicted}}-C_{\mathrm{actual}}}{)}^{2}=\frac{1}{N}\sum_{i=1}^{N}{\left(C_{\mathrm{predicted}\_i}-C_{\mathrm{actual}\_i}\right)}^{2}$ ; $\bar{C_{\mathrm{predicted}}}=\frac{1}{N}\sum {i=1}_{N}C_{\mathrm{predicted}\_i}$; $\bar{C_{\mathrm{actual}}}=\frac{1}{N}\sum {i=1}_{N}C_{\mathrm{actual}\_i}$.

### Field experiments for a new car

The experimental site was located at a specific institution in Beijing, China. Based on historical weather record, we conducted field experiments over 12 days. We processed data on 7 high-temperature workdays, from 2022 July 21 to 22, and 2022 July 25 to 29, with each day's measurements occurring approximately from 8:30 AM to 5:00 PM. The weather conditions during the measurement period contained sunny, cloudy, and rainy days (shown in Fig. 1). This facilitated our observation on the VOC emissions from the new car under diverse weather scenarios. The car used for tests was a hybrid electric car, and was about one month old at the time of experiment. The car remained in an engine-off stationary state during the experiment to avoid interference from other emissions from the car. All windows, doors, and operable air intakes were closed. Figure S3 illustrated the schematic for VOC measurement in the car. During the entire experiment, we placed a temperature and relative humidity data logger inside the car to facilitate continuous monitoring of the IAT and relative humidity. We measured the MST inside the cabin using a multichannel temperature recorder. To mitigate the influence of factors like solar radiation, thermocouple probes were positioned on the inner surface of the materials. The out-cabin air temperature was also measured by this instrument. At each sampling point, VOCs (excluding aldehydes and ketones) were initially sampled, followed by aldehydes and ketones. The sampling duration was 20 min, with a sampling interval of approximately 80 min, and the sampling flow rate was 500 mL/min. Aldehydes and ketones were sampled using DNPH sampling tubes and quantified by high-performance liquid chromatography (HPLC). VOCs (excluding aldehydes and ketones) were sampled using Tenax-TA sampling tubes and quantified via gas chromatography/mass spectrometry (GC/MS). Sampling locations were positioned approximately within the driver's breathing zone, and two collection tubes were used for simultaneous sampling at each instance. The average value was taken as the final concentration result. The pipelines of measuring and sampling equipment located outside the car were enveloped by insulating materials. These pipelines entered the cabin through the gap in the front windows, and the gap was sealed with nonemission aluminum foil tape. Measurements of cabin ventilation were also conducted in this closed condition.

## Supplementary Material

pgae243_Supplementary_Data

## Data Availability

All study data are included in the article and supporting information.
