# Supplementary material for: Cabin air dynamics: Unraveling the patterns and drivers of volatile organic compound distribution in vehicles
Source: PNAS Nexus. 2024 Jul 23;3(7):pgae243. doi: 10.1093/pnasnexus/pgae243 (PMC11264407; doi:10.1093/pnasnexus/pgae243)
Supplement: pgae243_Supplementary_Data [file pgae243_supplementary_data.pdf]

PNAS nexus

Supplementary Information for

Cabin air dynamics: Unraveling the patterns and drivers of VOC distribution in vehicles

Rui Zhang[1], Minglu Zhao[2], Hengwei Wang[1], Haimei Wang[1], Hui Kong[1], Keliang Wang[1], Petros Koutrakis[3], Shaodan Huang[3,4,*], Jianyin Xiong[1,*]

[1] School of Mechanical Engineering, Beijing Institute of Technology, Beijing 100081, China

[2] School of Computer Science and Engineering, Macau University of Science and Technology, Macau 999078, China

[3] Department of Environmental Health, Harvard T.H. Chan School of Public Health, Boston 02115, United States

[4] Department of Occupational and Environmental Health Sciences, School of Public Health, Peking University, Beijing 100191, China

[*]Corresponding author.

[1] Jianyin Xiong. E-mail address: xiongjy@bit.edu.cn

[2] Shaodan Huang. E-mail address: shhuang@bjmu.edu.cn

This PDF file includes:

Supplementary text S1
Figures S1 to S3
Tables S1 to S3

Supplementary Information Text S1

Attention mechanism.

Attention is an essential cognitive function in humans, involving a top-down information selection mechanism to filter out vast amounts of irrelevant information. This process aims to improve the issue of information overload by rapidly identifying and prioritizing high-value information. The attention mechanism enhances the weights of specific parts of input data while attenuating others, thereby directing the network's concentration towards a minute but critical subset of the data. This facilitates the extraction of detailed information related to the target of interest while concurrently dampening irrelevant information.

The attention layer has the capability to efficiently capture both global and local connections. Following this, data is unfolded via a dense layer to achieve the desired data dimensions, ultimately leading to the output stage through the output layer. This framework combines the strengths of the LSTM model, which inherently incorporates temporal information, and the attention mechanism. Building upon the LSTM model, the attention mechanism reorganizes the entire sequence, with a particular focus on the initial data. This reorganization facilitates a more rational search and evaluation of global information.

The calculation formula of the attention layer can be expressed as:

$$s_i = \tanh(Wh_i + b_i) \tag{1}$$

$$a_i = SoftMax(s_i) \tag{2}$$

$$V_i = \sum_{i=1}^{k} a_i h_i \tag{3}$$

where, h_i stands for the output of the LSTM hidden layer; s_i represents the similarity score; W and a_i are the weight matrix and weight coefficient, respectively; V_i represents the attention value.

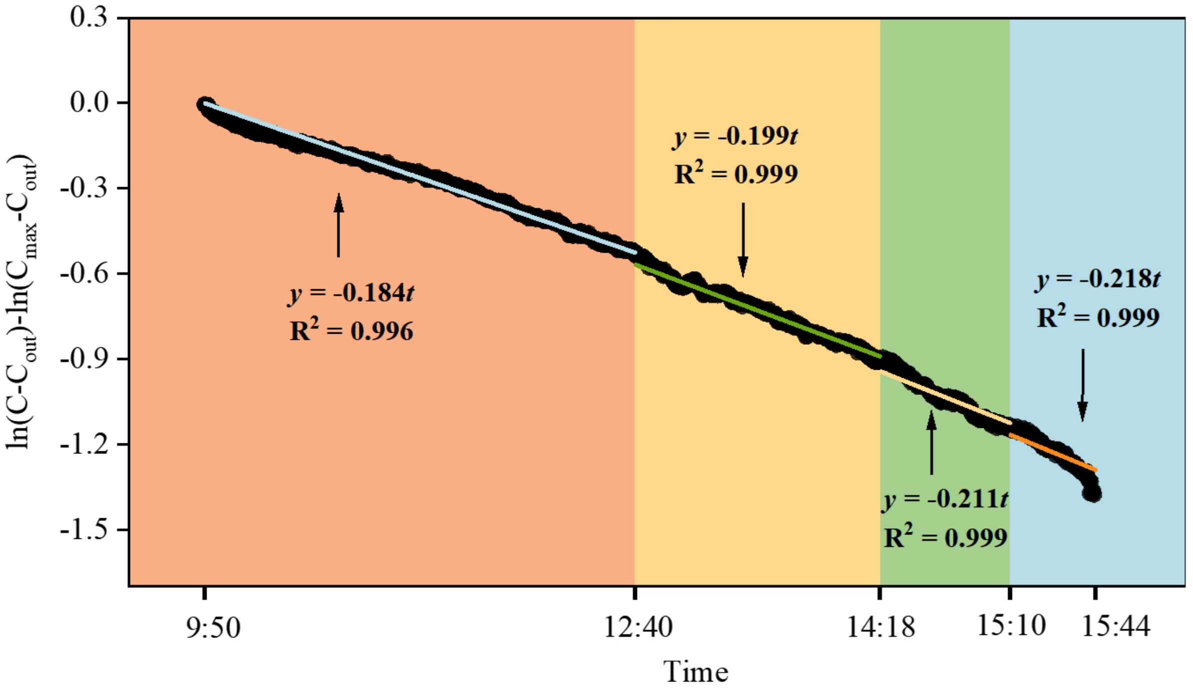

Fig. S1. AER of the stationary closed car cabin.

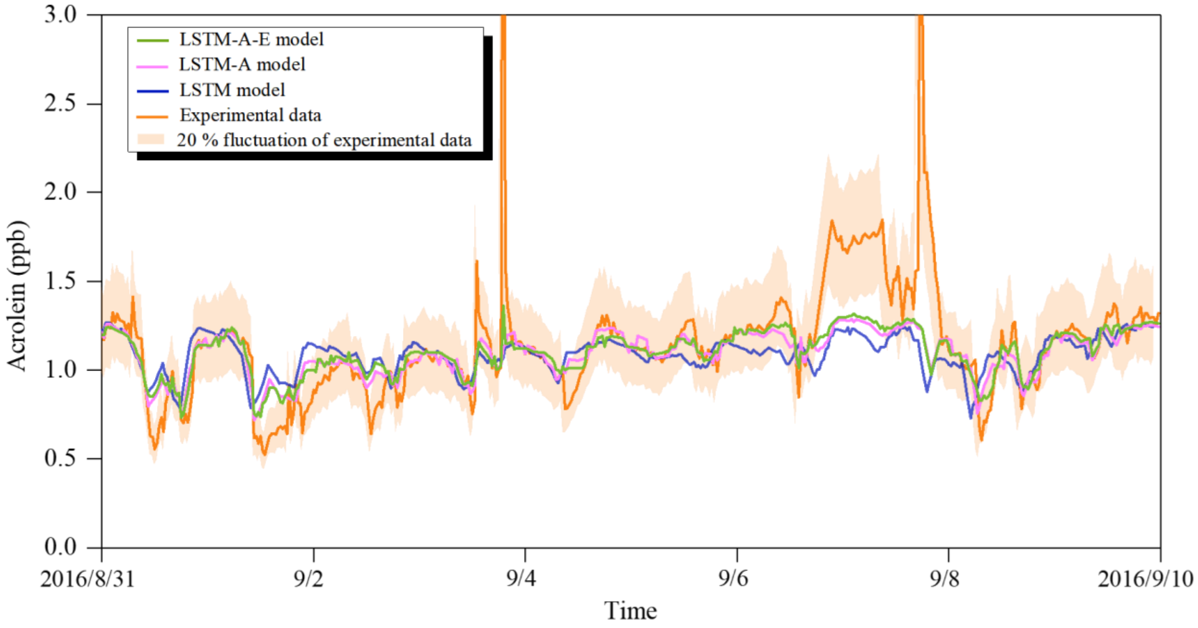

Fig. S2. Application of LSTM, LSTM-A, and LSTM-A-E models in predicting 10-day acrolein concentrations in a realistic residence.

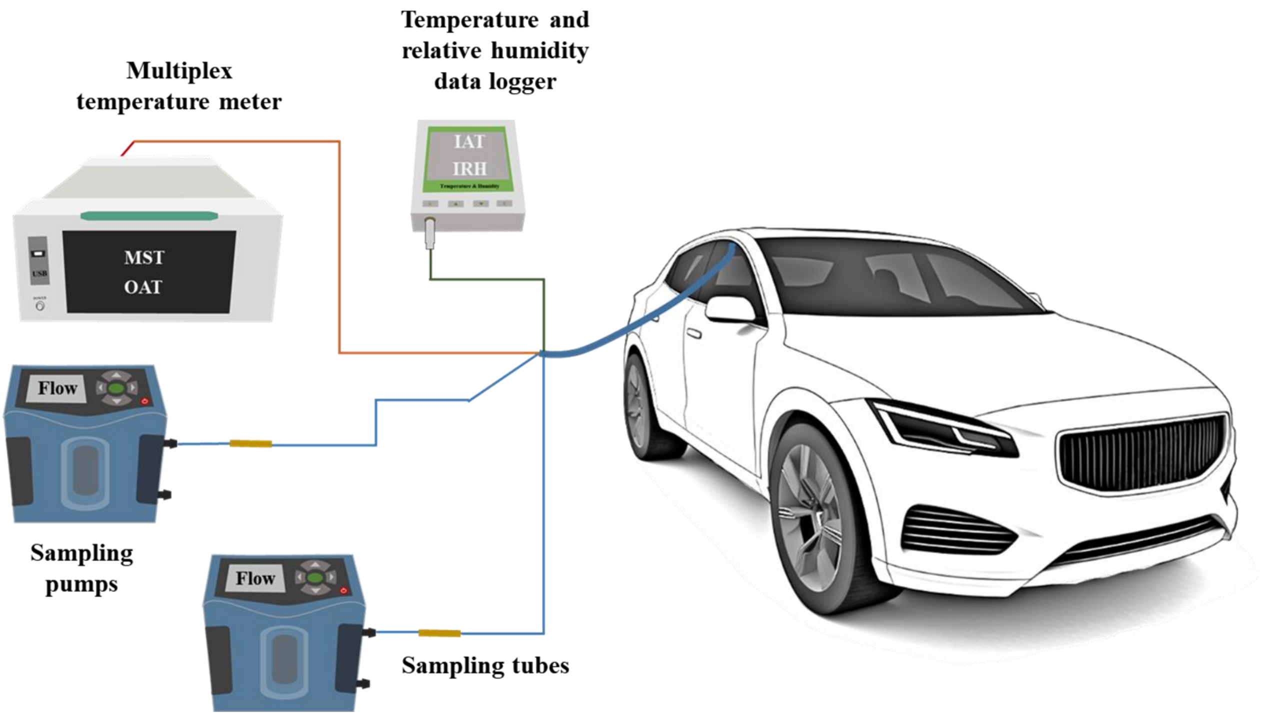

Fig. S3. Schematic of field measurements for the new car.

Table S1. Parameters for FNN, RNN, GRU, LSTM, LSTM-A and LSTM-A-E models.

Model	FNN	RNN	GRU	LSTM	LSTM-A	LSTM-A-E		
						LSTM-A1	LSTM-A2	LSTM-A3
Layers	2	2	2	2	2 LSTM+ 1 Attention	2 LSTM+ 1 Attention	2 LSTM+ 1 Attention	3 LSTM+ 1 Attention
The number of neural units per layer	128	128	128	128	128	128	256	128
Activation function	ReLU	ReLU	ReLU	ReLU	ReLU	ReLU	ReLU	ReLU
Dropout	0.1	0.1	0.1	0.1	0	0	0.1	0.1
Optimizer	Adam	Adam	Adam	Adam	Adam	Adam	Adam	Adam
Learning rate	0.001	0.001	0.001	0.001	0.001	0.001	0.001	0.001
Loss function	MSE	MSE	MSE	MSE	MSE	MSE	MSE	MSE
Iterations	250	250	250	250	250	250	250	250

Table S2. SSE, RMSE, NMSE, MAE, and MAPE values of the LSTM, LSTM-A and LSTM-A-E models for predicting acrolein concentration. SSE = The sum of squares due to error, ppb^2; RMSE = Root mean squared error, ppb; NMSE = Normalized mean square error; MAE = Mean absolute error, ppb; MAPE = Mean absolute percentage error.

Metrics	LSTM	LSTM-A	LSTM-A-E
SSE	60.386	49.223	45.521
RMSE	0.355	0.320	0.308
NMSE	0.102	0.081	0.075
MAE	0.175	0.126	0.121
MAPE	14.3%	9.8%	9.6%

Table S3. Model, range and accuracy for experimental testing instruments.

Instrument	Model	Range	Accuracy
Temperature and relative humidity data logger	TH20R (Miaoxin, China)	T: -40 ~ 80 °C<br>RH: 0 ~ 100%	T: 0.2 °C<br>RH: 2%
Multiplex temperature meter	TCP-24X (Huipu, China)	-999.99 ~ 9999.99 °C	High temperature:<br>0.5% + 0.6 °C<br>Low temperature:<br>0.5% + 1.5 °C
Sampling pumps	Defender 520	50 ~ 5000 ccm	1%
Indoor air quality meter	CA 1510 (Chauvin Arnoux Metrix, France)	0 ~ 5000 ppm	50 ppm
